# Phenology and Growth dynamics of *Avicennia marina* in the Central Red Sea

**DOI:** 10.1038/srep37785

**Published:** 2016-11-28

**Authors:** Hanan Almahasheer, Carlos M. Duarte, Xabier Irigoien

**Affiliations:** 1 King Abdullah University of Science and Technology (KAUST), Red Sea Research Center, Thuwal 23955-6900, Kingdom of Saudi Arabia; 2 Biology Department, University of Dammam (UOD), Dammam 31441-1982, Kingdom of Saudi Arabia

## Abstract

The formation of nodes, stem elongation and the phenology of stunted *Avicennia marina* was examined in the Central Red Sea, where *Avicennia marina* is at the limit of its distribution range and submitted to extremely arid conditions with salinity above 38 psu and water temperature as high as 35° C. The annual node production was rather uniform among locations averaging 9.59 node y^−1^, which resulted in a plastocron interval, the interval in between production of two consecutive nodes along a stem, of 38 days. However, the internodal length varied significantly between locations, resulting in growth differences possibly reflecting the environmental conditions of locations. The reproductive cycle lasted for approximately 12 months, and was characterized by peak flowering and propagule development in November and January. These phenological observations provide a starting point for research and restoration programs on the ecology of mangroves in the Central Red Sea, while the plastochrone index reported here would allow calculations of the growth and production of the species from simple morphological measurements.

Mangroves are woody trees and shrubs that occupy the intertidal zone in the tropics and subtropics^1^,^2^,^3^, where they contribute to primary production^4^ and provide a range of ecosystem services^5^. Forming lush vegetation in the wet tropics, mangroves are often stunted in regions with limited freshwater runoff, including karstic^6^ and arid^7^ regions.

*Avicennia marina* has the widest latitudinal as well as longitudinal distribution among mangroves^3^. Thus *Avicennia marina* occupies coastal areas across a broad range of environmental conditions, therefore suggesting that the species must have considerable growth plasticity^8^. *Avicennia marina* is the dominant mangrove species in the Red Sea^9^, where its growth is limited due to lack of rivers and freshwater input, low nutrient supply, high salinity, and hot summer air temperature^10^. Indeed, *Avicennia marina* are stunted in the Central Red Sea (about 2 to 3 m in height), compared to 16 m tall trees reported in Australia^11^.

The Red Sea represents, therefore, an end member in terms of the combination of growth conditions to support *Avicennia marina* growth. However, the phenology and growth dynamics of *Avicennia marina* in the Red Sea has only been assessed in a single study, reporting the reproductive cycle of the Southern Red Sea Coast of Saudi Arabia^12^. Indeed, there is a paucity of information on the phenology of the species, with a limited number of reports worldwide^13^,^14^,^15^,^16^,^17^,^18^,^19^,^20^,^21^,^22^. This is a surprising gap, as this species plays a major role as a source of food and nursery grounds, as an intense carbon sink and in offering coastal protection in tropical coastlines around the world^23^. The harsh conditions in the Red Sea, particularly in summer, may affect the phenology and growth of *Avicennia marina,* which may deviate from those reported elsewhere.

Here we report the phenology and growth dynamics of monospecific stands of stunted *Avicennia marina* in the Central Red Sea. In particular, we estimate their annual growth, including the node and branch production as well as the phenology of their reproductive stages: budding, flowering, and fruiting. The phenology of this species is an essential biological trait required to underpin ecological research on this important species and to plan restoration projects, as well as to provide a baseline to assess responses to climate change^24^. Whereas the internodal production of the congeneric species *Avicennia alba* has been reported for SE Asia at 17.6 nodes per year^25^, that of *Avicennia marina* has not been reported anywhere along its extended range.

## Results

**Figure 1:**
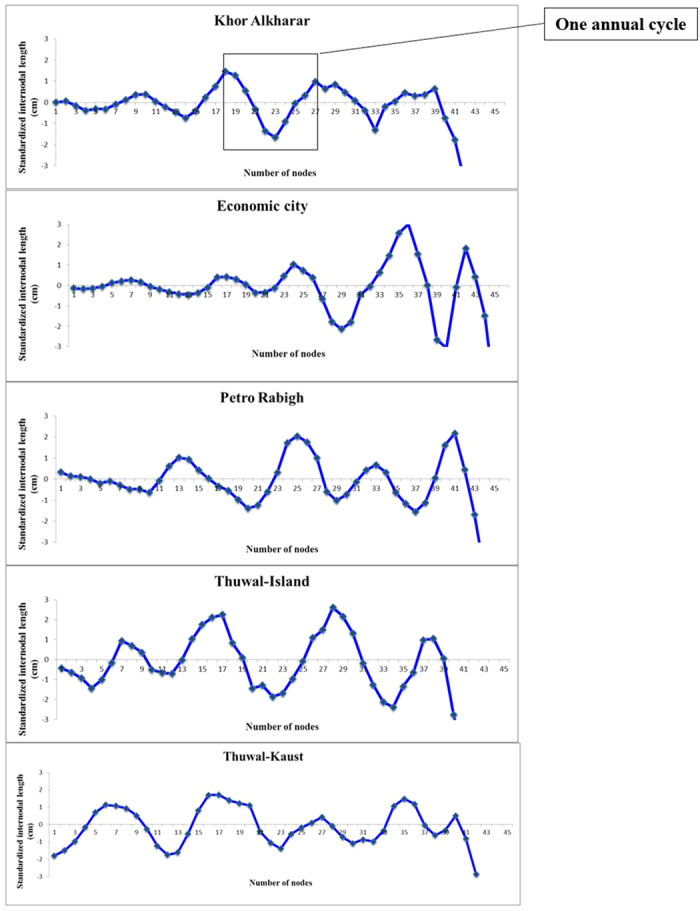
Examples of standaraized internodal length for a selected plants in each study site in the Central Red Sea.

**Figure 2:**
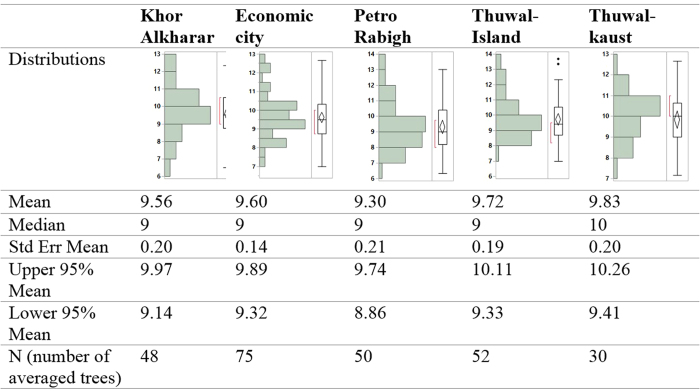
Number of nodes produced by the main axis of *Avicennia marina* trees (nodes/year) in five stands sampled in the Central Red Sea.

**Figure 3:**
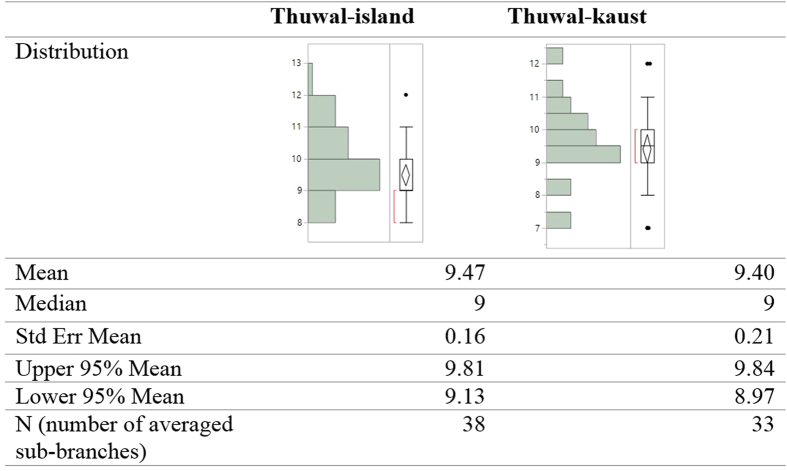
Number of sub-branches produced annually by *Avicennia marina* trees in two stands sampled in the Central Red Sea (Thuwal).

All of the examined trees showed very clear interannual cycles of internodal length along their main axis when the data were filtered to remove long-term and short-term variability (Fig. 1). The node production ranged from 9.3 to 9.8 node y^−1^ and did not differ significantly among populations (F = 0.93 and P = 0.44, Fig. 2), resulting in a range of plastochrone intervals of 39.20 to 37.90 days. There were no significant differences in the number of internodes produced per year between locations (Tukey HSD post hoc test, P < 0.05, Fig. S1), which averaged 9.59 ± 0.08, N = 255 across stands, corresponding to an average plastochrone interval of 39 days. This result was validated by the results derived from the seedlings grown in the nursery, for which the plastochrone interval was calculated to be 38 days. Further, validation was provided by the annual production of total sub-branches in the axillary shoot, which produced on average 9.44 ± 0.13 nodes sub-branch y^−1^ (N = 71), corresponding to a plastochrone interval of 39.20 ± 0.56 days, without significant differences between the two locations (F = 0.0576, P < 0.0001 and Student’s *t*-test, P < 0.05, Fig. 3, Fig. S2).

**Figure 4:**
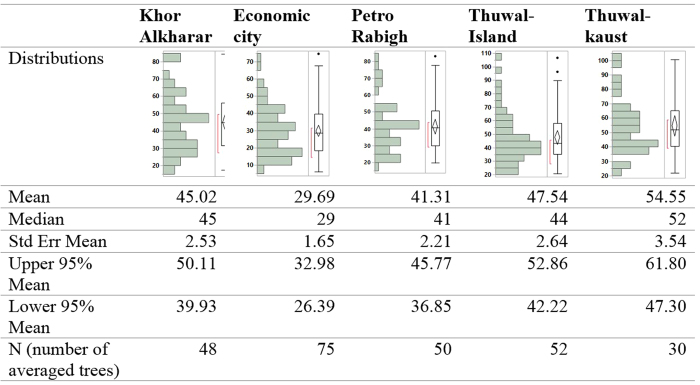
Elongation rate (cm y^−1^) of the main axis of *Avicennia marina* trees in five populations sampled in the Central Red Sea.

In contrast to the annual node production the internodal length varied significantly between locations (F = 15.85 and P < 0.0001, Fig. 4), with plants at Thuwal-Kaust developing significantly longer internodes, followed by both Thuwal-Island and Khor Alkharar, then Petro Rabigh, and finally Economic city (Tukey HSD post hoc test, P < 0.05, Fig. 4, Fig. S3). As a result, annual mangrove stem elongation varied across sites (F = 9.10 and P < 0.0001), with a higher annual growth in Thuwal-Kaust, followed by Thuwal-Island, Khor Alkharar and Petro Rabigh, and then the lowest growth in Economic city (Tukey HSD post hoc test, P < 0.05, Table 1). However, no significant differences in node production or internodal length were observed over the years where these properties were regenerated (F = 0.66 and P = 0.5745).

**Figure 5:**
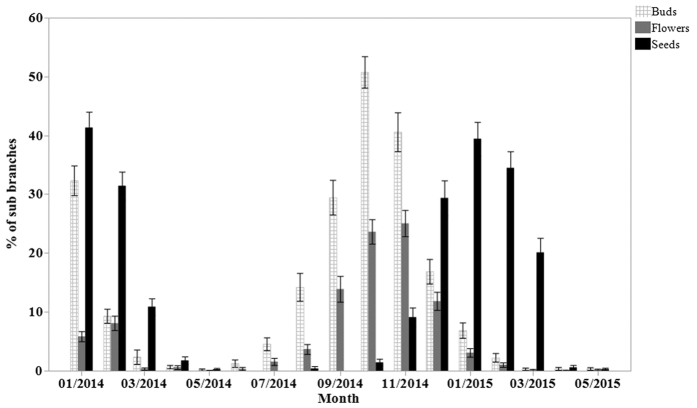
Phenology patterns and mean percent reproductive branches in a *Avicennia marina* populations in the Central Red Sea (Thuwal).

**Figure 6:**
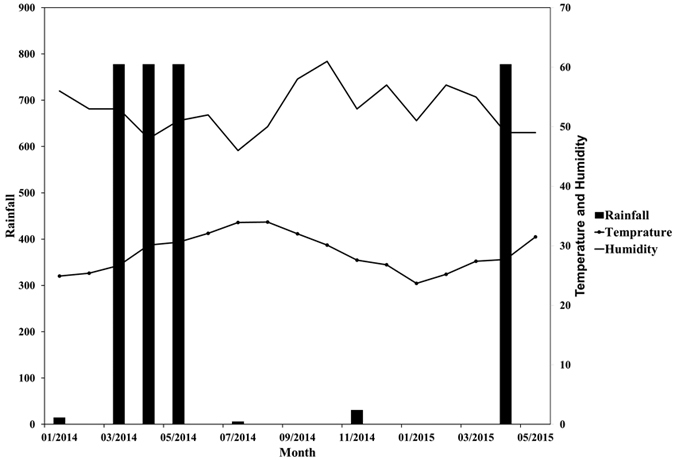
Climate diagram for temperature, humidity and rainfall in the Central Red Sea.

Bud production was initiated in June and the reproductive cycle concluded with the release of propagules in April (Fig. 5), thereby elapsing over a total 10 months. Propagules development lagged flowering by about 3 months, which is the approximate time elapsed from pollination to propagules maturation (Fig. 5). The buds peaked in October with an average (±SE) of 50.8 ± 5.1% of the sub-branches producing them, followed by a flowering peak in November, with 25.1 ± 4.4% productive sub-branches flowering. Propagules production peaked in January, with 39.4 ± 5.0% productive sub-branch (Fig. 5). Bud and flower production are initiated at the time of the summer solstice (20–23 June), when temperature is highest, but the peak of buds and flower production occur in the Autumn, when atmospheric humidity is highest (Fig. 6).

## Discussion

The stem elongation of *Avicennia marina* seedlings provides a record of internodal length that allows the reconstruction of its growth patterns and calculation of the plastochrone interval, a key property to convert biological into chronological time^26^, consistent with findings for other mangrove species^25^. The annual node production estimated from the analysis was 9.59 nodes y^−1^, corresponding to an average plastochrone interval of 38 days. This estimate is consistent with that derived from the observation of annual sub-branch production of 39 days, and that directly observed for seedlings growing in a nursery of 37 days. Hence, the 38 days plastochrone interval provides, therefore, a robust estimate to convert number of internodes into time elapsed, which can be used to derive mangrove seedling stem elongation and production. This is important as the plastochrone interval for *Avicennia marina* had not been resolved to date, despite being a key mangrove species with a broad distribution^27^. The production of 9.59 nodes y^−1^ for *Avicennia marina* in the Central Red Sea, is within the range of reports for other species, spanning from 3.8 nodes y^−1^ for *Rhizophora* seedlings in Panama^28^ to 30.3 nodes y^−1^ for *Sonneratia caseolaris in* Thailand^29^ (Table 2). Moreover*, Avicennia marina* node production is below that for the cognatic Asian species *Avicennia alba* (Table 2).

Contrary to node production, which was conserved across *A. marina* stands in the Central Red Sea, the internodal length varied between locations, resulting in annual changes in stem growth and production. Whereas the plastochrone interval is believed to be under genetic control^26^, as supported by the absence of significant differences among the stands studied here, the plasticity in the length of internodes across stands suggests that this trait is under environmental control, possibly including freshwater inputs and nutrient limitation, which contributes to the dwarfing of *Avicennia*^30^, such as that the species displays in the Red Sea. Indeed, recent experiments found iron to be limiting the growth of *A. marina* seedlings in Central Red Sea^31^. Duke^14^ reported that the timing and success of *Avicennia* reproductive cycle increase by a factor of two or three for each additional 10 °C up to a maximum temperature of about 34 °C. Therefore the low growth rates in the Red Sea are unlikely to be attributable to high temperature alone. Either temperature or photoperiod determines the onset of the budding and flowering season, as increasing temperature with peak solar radiation triggers flower development and high humidity is conducive to propagules development. Confirming the role of photoperiod, temperature and humidity in *A. marina* phenology requires, however, experimental validation, as these are strongly correlated in the field. In addition, seawater salinity increases from the south at about 36.5 psu near the strait of Bab el Mandeb, to the north at about 41–40 psu, at the southern tip of the Sinai Peninsula^32^. Yet, the salinity in a coastal lagoon near Jeddah varied between 40.5 psu in April and 41.8 psu in August^33^. Therefore, the low growth rate might be attributed to the high salinity as well.

Even though tree production may vary interannually, there seems to be a balanced breeding system keeping a productive population with strong recruitment and dispersal potential^16^. The variability in the reproductive branches of *A. marina* in Central Red Sea (i.e. when comparing Jan 2014 and Jan 2015) is typical for plants, where individual branches do not flower in consecutive years but branches in the same tree are offset in the biannual cycle so the tree flowers and produces propagules every year^13^. Fruits follows a similar pattern which suggests a limitation in the number of shoot axes able producing them^15^.

The duration of the reproductive cycle of *A. marina* in this study at 22°N (approximately 12 months) is similar to that of *A. marina* in the southeast coast of Australia (approximately 12 months) at 35°S^16^, and longer than the reproductive cycle reported in Kenya at 4°S, where bud initiation to fruit falling lasted 6 to 8 months^21^. Because generally flowering/fruiting cycles are connected to latitude^34^ with the floral initiation being later with increasing latitude^15^ it has been suggested that the reproduction of *Avicennia marina* is less successful at higher latitudes because of the shorter summer decreasing the growth period^14^.

In conclusion, *A. marina* mangroves in the Central Red Sea produce about 9.59 nodes y^−1^ per axis and have a reproductive period of 12 months. The node production did not vary between locations whereas internodal length varied significantly between locations, resulting in growth differences probably reflecting the environmental conditions of each location (i.e. the limitation in nutrient supply and fresh water inputs). The results presented on the basic phenology of *Avicennia marina* in the Central Red Sea provide an important baseline for research on the ecology of the species and is, therefore, an important resource for many applications, including planning of restoration projects, which require the availability of propagules and, therefore, should be informed by knowledge of the reproductive phenology of the species. In addition, the plastochrone index of *Avicennia marina* in the Central Red Sea reported here would allow calculations of the growth and production of the species from simple morphological assessments, as demonstrated by Duarte *et al*.^25^, therefore allowing rapid assessments of growth conditions and plant performance for monitoring purposes.

## Methods

### Internodal measurements in Central Red Sea mangroves

**Figure 7:**
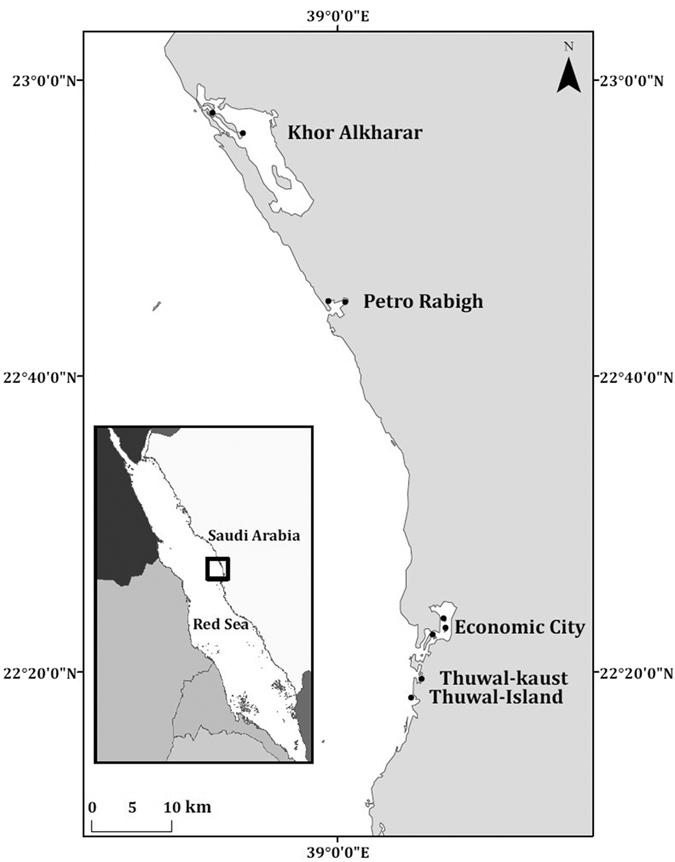
Study sites in the Central Red Sea. The sites are located in the kingdom of Saudi Arabia. The map was produced with ArcMap Version 10.2. Background map credits: the World Administrative Divisions layer provided by Esri Data and Maps, and DeLorme Publishing Company. Redistribution rights are granted http://www.esri.com/~/media/Files/Pdfs/legal/pdfs/redist_rights_103.pdf?la=en.

Measurements of internodal length sequences, which allow inferences on the growth dynamics of mangroves^26^, were conducted in five contrasting Central Red Sea mangrove stands, supporting different levels of human impacts (Fig. 7). Mangroves in the Thuwal area were cleared by the construction of the King Abdullah University for Science and Technology (KAUST) in 2007 and there was a new plantation program in 2010 that allows us to validate the age estimates of the mangroves. Both Thuwal-Island and Khor Alkharar are areas undisturbed by human activities. On the contrary, Petro Rabigh suffers industrial impacts from petrochemical activities and the Economic city area is impacted by the construction of the city (Fig. 7).

**Figure 8:**
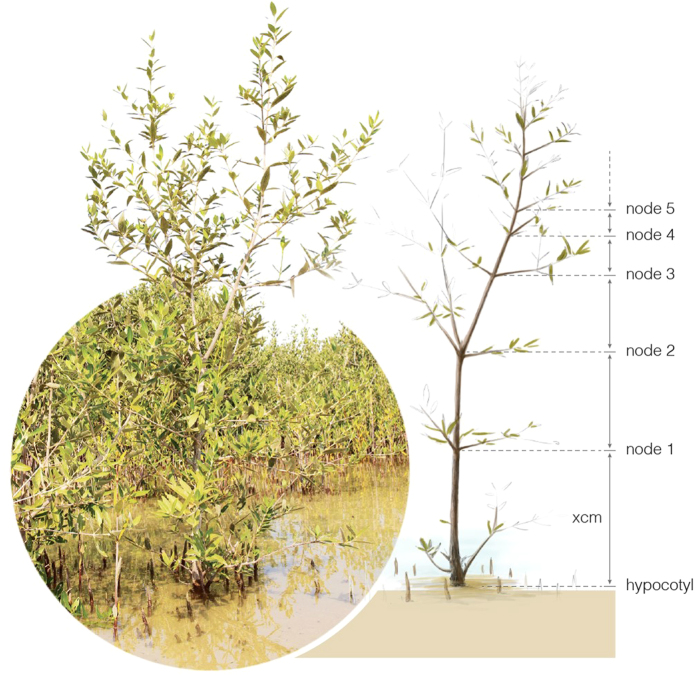
An illustration of counting nodes and measuring internodal length. Photo by H.A and the artist work by I. Gromhico.

We reconstructed mangrove growth dynamics based on the cycles in internodal length^25^. To study internodal growth, we selected 48 trees in Khor Alkharar, 75 trees in Economic city, 50 trees in Petro Rabigh, 52 trees in Thuwal-Island and 30 trees in Thuwal-Kaust (total, n = 255 tree) to assess the internode production and length and derive annual growth. The main criteria to select the trees was the possibility to clearly follow the main axis. Where possible we counted and measured the nodes along the main axis from the apical meristem to the node that could be safely identified nearest to the hypocotyl (Fig. 8). Overall, the average height of the selected trees was 184.7 cm with a size distribution ranging from 105 cm to 370 cm. The internodal series measured for individual trees assessed averaged 33 internodes, with a maximum of 51 internodes in Thuwal-Kaust and a minimum of 17 internodes in Khor Alkharar.

Mangrove plants have been shown to produce a fixed number of nodes annually, and to display an annual cycle of internodal length that can be used to discern the number of nodes they produce annually from records of internodal length along the main axis of mangrove branches and saplings using the reconstruction technique formulated by Duarte *et al*.^25^. Because multiple effects have been found to add noise to the sequences of internodal lengths in mangrove trees resulting in both short term (e.g. storms) or long term (e.g. interannual variations)^25^, we applied a long-pass and a short-pass filter to remove such noise for the internodal measurements, thereby highlighting variability at annual scales. The long-term filter involved subtracting the sequences of raw internodal length measurements from values corresponding to a running average of n = 14 internodes centered at the specific node being filtered to remove long term variability. Then the resulting values were filtered for short-term noise by subtracting a running average of n = 3 internodes centered at the specific node being filtered. For fifty (out of 255) trees the n = 3 or n = 4, depending on the amount of short-term noise, the node production was calculated for each location after counting the number of nodes between consecutive maxima along the filtered sequence and averaging the values for each tree (Fig. 1)^25^, and then averaging those for each population examined. The annual growth was calculated as the result of the sum of internodal lengths during each annual cycle. In general, the sequences of internodal lengths collected along stems allowed the identification of four to five phenological years, thereby also allowing to assess growth variability over the past 4 to 5 years.

### Estimating plastochrone interval

To directly examine the plastochrone interval (the average time in between the production of a node^26^), thirty *A. marina* propagules collected from Thuwal-Island were sprouted in February 2014 and grown in a nursery with brackish water for 91 days. We monitored the number of nodes produced over time by the seedlings grown in a nursery^31^. We then calculated the plastochrone interval (as the average ratio between the time elapsed and number of nodes produced) to validate the estimate derived from the reconstructive analysis of growth cycles.

### Monthly phenological observations

We monitored monthly between January 2014 and May 2015 a total of 96 axillary shoots distributed over 32 trees in two different stands 5 kilometers apart (Thuwal-Island and Thuwal-Kaust). These shoots were tagged and we counted the number of sub-branches with buds, flowers and propagules in each shoot, to link the *Avicennia marina* phenology to weather we used data from the Presidency of Meteorology and Environment (PME) in the Kingdom of Saudi Arabia. We also calculated the annual branching production from the time series of the number of branches along each of the tagged stems using the same filters that we used earlier for the node production in the main axis of the tree, because according to^28^ counting the nodes between apical meristem to the hypocotyl is often equal from the hypocotyl to the axillary shoot. Seventeen (out of 96) branches were excluded from the analysis because of failure to detect a clear annual growth cycle.

### Statistical analysis

Statistical analyses, including descriptive statistics, general linear models to test the differences between trees and locations, as well as Student’s *t*-test and Tukey HSD posthoc test to assess pairwise differences were carried out using JMP, a computer program for statistics developed by the SAS Institute.

## Additional Information

**How to cite this article**: Almahasheer, H. *et al*. Phenology and Growth dynamics of *Avicennia marina* in the Central Red Sea. *Sci. Rep.* **6**, 37785; doi: 10.1038/srep37785 (2016).

**Publisher's note:** Springer Nature remains neutral with regard to jurisdictional claims in published maps and institutional affiliations.

## Supplementary Material

Supplementary Information

## Figures and Tables

**Table 1:** Mean ± SE (n) of the internodal length (cm y^−1^) of the main axis of *Avicennia marina* trees over five consecutive phonological years in five populations sampled in the Central Red Sea.

Location	1^st (2014–2015)^	2^nd (2013–2014)^	3^rd (2012–2013)^	4^th (2011–2012)^	5^th (2010–2011)^	Average Total, cm
Khor Alkharar^a,b^	41.99 ± 2.75 (48)^a^	46.24 ± 3.68 (26)^a^	28.50 ± 4.87 (3)^a^	34.50 ± 13.00 (2)^a^		42.69 (79)
Economic city^c^	26.70 ± 1.71 (75)^a^	28.59 ± 2.17 (57)^a^	27.11 ± 2.71 (19)^a^	29.51 ± 7.59 (6)^a^		27.54 (157)
Petro Rabigh^b^	38.29 ± 2.37 (50)^a^	43.03 ± 2.98 (35)^a^	36.22 ± 5.10 (10)^a^	29.4 (1)^a^		39.71 (96)
Thuwal-Island^a,b^	43.85 ± 2.92 (52)^a^	45.09 ± 2.88 (43)^a^	50.29 ± 5.60 (15)^a^	52.63 ± 20.88 (2)^a^		45.34 (112)
^*^Thuwal-kaust^a^		49.35 ± 4.38 (30)^a^	48.44 ± 3.39 (26)^a^	54.56 ± 6.02 (13)^a^	44.75 ± 19.75 (2)^a^	49.84 (71)

The annual growth was calculated as the sum of internodal lengths produced during each annual cycle. The letters correspond to Tukey posthoc HSD multiple comparison testing for significant differences between years for each location, the same letters means no significant differences within one location (P > 0.05).

^*^The measurements in Thuwal-Kaust were conducted one year earlier than those at other locations.

**Table 2:** Reported number of nodes produced annually along the main axis by different mangrove species around the world.

Species	Nodes y^−1^	Location	Reference
*Avicennia alba* (transplanted)	13.2	Thailand	29
*Avicennia alba (*natural)	17.6	SE Asia	25
*Avicennia marina (*natural)	9.59	Central Red Sea	This study
*Avicennia marina (*natural)	9.44	Central Red Sea	This study branch y^−1^
*Avicennia marina* (planted)	9.63	Central Red Sea	This study
*Rhizophora* sp (planted)	5.4*	Caribbean coast of Panama	28nodes shoot^−1^ y^−1^
*Rhizophora* sp (natural)	5.5	Philippines	35
*Rhizophora apiculata* (natural)	7.3	SE Asia	36
*Rhizophora apiculate (*natural)	8.03	SE Asia	25
*Rhizophora mucronata* (transplanted)	6.5	Thailand	29
*Sonneratia caseolaris (*natural)	28.8	SE Asia	25
*Sonneratia caseolaris* (transplanted)	30.3	Thailand	29

^*^Reclculated from 3.8 ± 0.3 to 7.0 ± 0.6.
