# Supplementary material for: Phenology and Growth dynamics of Avicennia marina in the Central Red Sea
Source: Sci Rep. 2016 Nov 28;6:37785. doi: 10.1038/srep37785 (PMC5125269; doi:10.1038/srep37785)
Supplement: Supplementary Information [file srep37785-s1.pdf]

Phenology and Growth dynamics of *Avicennia marina* in the Central Red Sea

Hanan Almahasheer[1, 2], Carlos M. Duarte[1] and Xabier Irigoien[1, *]

[1] King Abdullah University of Science and Technology (KAUST), Red Sea Research Center, Thuwal 23955-6900, Kingdom of Saudi Arabia

[2] Biology Department, University of Dammam (UOD), Dammam 31441-1982, Kingdom of Saudi Arabia

*: Corresponding author: hanan.almahsheer@kaust.edu.sa

Table S1. Annual node production y^{-1} for each individual interannual cycle

	**Alkarrar**	**Economic-city**	**Rabigh**	**Thuwal-island**	**Thuwal-kaust**
Distribution	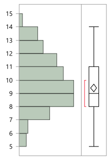	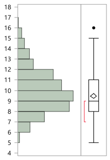	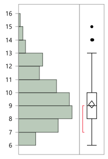	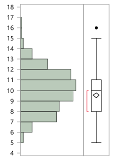	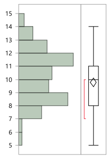
Mean	9.40	9.46	9.10	9.53	9.77
Median	9	9	9	10	10
Std Err Mean	0.15	0.11	0.14	0.12	0.16
Upper 95% Mean	9.70	9.69	9.38	9.79	10.10
Lower 95% Mean	9.09	9.24	8.81	9.28	9.44
N	166	321	182	226	139

Table S2. Sub branching production for one single interannual cycle

	**Thuwal-island**	**Thuwal-kaust**
Distribution	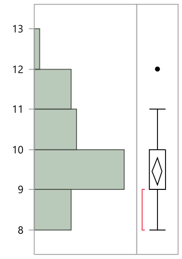	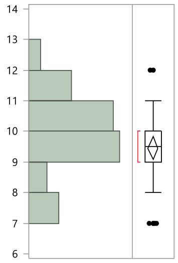
Mean	9.45	9.45
Median	9	9.5
Std Err Mean	0.16	0.18
Upper 95% Mean	9.78	9.83
Lower 95% Mean	9.11	9.07
N	40	46

Table S3. Internodal length y^{-1} for each single interannual cycle

	**Alkarrar**	**Economic-city**	**Rabigh**	**Thuwal-island**	**Thuwal-kaust**
Distribution	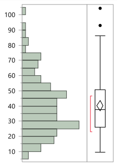	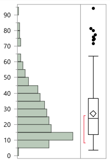	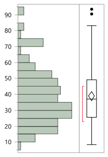	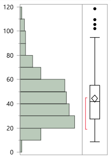	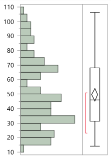
Mean	40.36	26.92	38.95	44.39	49.56
Median	37.75	23.9	37.1	42	45.8
Std Err Mean	1.68	1.01	1.44	1.51	2.10
Upper 95% Mean	43.70	28.91	41.79	47.37	53.72
Lower 95% Mean	37.02	24.92	36.10	41.40	45.41
N	130	266	153	191	123
